# Functional Aspects of Fish Mucosal Lectins—Interaction with Non-Self

**DOI:** 10.3390/molecules23051119

**Published:** 2018-05-09

**Authors:** Monica Fengsrud Brinchmann, Deepti Manjari Patel, Nevil Pinto, Martin Haugmo Iversen

**Affiliations:** 1 Faculty of Biosciences and Aquaculture, Nord University, 8049 Bodø, Norway; deepti.m.patel@nord.no (D.M.P.); nevilpinto1@gmail.com (N.P.); martin.h.iversen@nord.no (M.H.I.); 2 Department of Fish Genetics and Biotechnology, ICAR-Central Institute of Fisheries Education, Mumbai 400061, India

**Keywords:** lectin, teleost, mucosal, innate immunology, antibacterial, phagocytosis, agglutination

## Abstract

Mucosal surfaces are of key importance in protecting animals against external threats including pathogens. In the mucosal surfaces, host molecules interact with non-self to prevent infection and disease. Interestingly, both inhibition and stimulation of uptake hinder infection. In this review, the current knowledgebase on teleost mucosal lectins’ ability to interact with non-self is summarised with a focus on agglutination, growth inhibition, opsonisation, cell adhesion, and direct killing activities. Further research on lectins is essential, both to understand the immune system of fishes, since they rely more on the innate immune system than mammals, and also to explore these molecules’ antibiotic and antiparasitic activities against veterinary and human pathogens.

## 1. Introduction

Lectins are proteins that bind to carbohydrate, proteoglycans, glycolipids, and glycoproteins. The sources of lectins from marine environments are diverse and a review focused on their structure, evolution, and therapeutic potentials can be found in Reference [1], and comprehensive reviews on fish lectins are found in References [2,3,4].

Lectins are classified based on the specificity of their carbohydrate binding domain and dependence on divalent cations for their activities [2]. The major classes of fish lectins include C-type lectins, F-type lectins, galectins, rhamnose-binding lectin, ricin-type, lily-type, and 6x β-propeller/tectonin-type lectins [1,4]. They have a wide array of functions including immune relevant ones such as pathogen recognition, agglutination, opsonisation, complement activation and phagocytosis, and other functions such as splicing of RNA, protein folding, trafficking of molecules, and control of cell proliferation [2,4]. Lectins are found both intracellularly and extracellularly in biological fluids such as serum and mucus. In fish, several lectins with possible immune functions have been isolated from mucosal surfaces suggesting an important role in defence in the mucosa.

Mucosal surfaces have living epithelial cells throughout the surface and are covered by mucus. In fish the eyelids, nose, mouth, gastrointestinal tract, and bladder are mucosal surfaces, as in mammals. In addition, in fish, the skin and gills are mucosal surfaces. The mucosal tissues on the surface of fish are constantly exposed to water where viruses, microorganisms, and parasites, as well as abiotic factors, are present. This puts the fish at risk, and to remain in homeostasis and healthy, the mucosal surfaces must have a strong immunological defence system. The skin mucosal surface is the largest mucosal surface in fish. In recent years -omics technologies have established a knowledge base related to the molecules present in skin mucus [5] and teleost mucosal surfaces [6]. Limited information is available on the molecular interaction of bacteria with mucosal surfaces, including the skin [7]. Information is also scarce on the mechanisms of interaction between teleost mucosae and viruses, yeast, and other eukaryotic organisms. Knowledge in these areas is important to understand the mucosal surfaces’ roles as biological barriers against pathogens, and the interaction with commensal organisms. Work on gnotobiotic (germ-free) zebrafish (*Danio rerio*) has clearly shown that the gut microbiome is crucial for the normal function of the mucosa in biological processes such as nutrient uptake, immunological function, and epithelial renewal [8].

Mucosal lectins from fish have been isolated from mucus and tissues or recombinantly expressed for in vitro studies with pathogens. Antibodies against lectins have been developed and they have identified lectins on or close to pathogens in histological sections or in binding studies. This review summarises the progress made in lectin biology related to teleost mucosal lectins’ interaction with non-self and points out needs for further research. Pathogen binding lectins are interesting not only for fish biologists, but also have the potential to be used in veterinary and human medicine.

In this review the current knowledge on teleost mucosal lectins that can interact with non-self, including pathogens and commensals, is discussed in a functional perspective mainly based on in vitro studies because of limited in situ studies on whole animals and limited host in vivo cell culture studies.

## 2. Localisation of Lectins in Mucosae

Lectins are found both intracellularly and extracellularly in the mucus. They are secreted into the mucus by several pathways such as the classical endoplasmatic reticulum (ER)/Golgi apparatus secretory pathway, where the protein is synthesised on the ER co-translationally and is eventually transported to the cell surface. Other mechanisms are through non-classical transport including through channels or by transporters, exosomes, and microvesicles [9,10]. Proteins secreted from the ER-Golgi pathway are in general glycoproteins, and have carbohydrates added to them through N-linked glycosylation and O-linked glycosylation in the ER and Golgi, respectively. The mannose-binding lectin pufflectin, from the skin mucus of the pufferfish (*Fugu rubripes*) appears not to be glycosylated, suggesting a non-classical secretion mechanism [11]. Natterin-like protein from Atlantic cod (*Gadus morhua*) does not have a signal peptide for binding to signal recognition particles, and hence cannot be secreted through the ER-Golgi pathway. As it is found extracellularly in skin mucus [12,13], a non-classical secretion mechanism is suggested [13]. The galectins are also lectins that show a non-classical secretion mode [9,14].

Lectins can be localised on cell surfaces, in cytosol, and in intracellular organelles. Epithelial galectins can be localised in the nucleus where they are involved in RNA splicing, in endosomes where they play a role in endosome trafficking; in addition, galectin members can partly show differences in tissue distribution (review on epithelial galectins in [15]). Galectins can show differences in cellular distribution within the tissues. In Atlantic cod (*Gadus morhua*), antibodies against the two homologs Codgal1-1 and Codgal1-2 labelled different cells in the skin, gill, and intestine. Codgal1-1 was found in the epithelial layer in the skin, gills, and rectum, and in addition in basal cells in the skin and endothelial cells in the gills. The closely homologous Codgal1-2 was found in epithelial cells and basal cells in the skin just like Codgal1-1. However, Codgal1-2 was found in other cells than Codgal1-1 in the gills and rectum. In the gills, blood leukocytes were Codgal1-2 positive and in the rectum, Codgal1-2 was found in macrophage-like cells [16]. Galectin from Atlantic cod is also found in skin mucus [17]. This shows that in a species different tissues and different cells can express galectins.

In the European eel (*Anguilla anguilla*)， seven fucolectins were found. Two were further studied and showed differential expression. One gene was expressed in the liver for secretion of the translated protein into the serum, whilst another was expressed in the gills and likely had a role in mucosal protection [18]. Therefore, several homologues of a lectin can be present in a species, these can show tissue and cell specific expression, and in addition, differences in expression can be found in different developmental stages of the fish. For example, *codgal1-1* is expressed in fertilised eggs, the early cell division stages until first feeding and in the adult fish. *codgal1-2* is also expressed in fertilised eggs, but is weakly expressed in the early cell divisions until the 30 somite stage where expression becomes strong until the first feeding up to adult [16].

AJL-2, the C-type like lectin from the Japanese eel (*Anguilla japonica*) is found both in the skin mucus and in club cells in the skin [19]. The galectin AJL-1 from the Japanese eel is found in the skin and mucus, and the gene is only expressed in the skin [20]. Interestingly, in adult tropical eel (*Anguilla marmorata*), only one lectin was found, an orthologue of AJL-1 [21]. In juvenile tropical eel on the other hand, gene and protein expression of an orthologue of the lectin AJL-2 were found in the epidermis [21].

Taken together it is clear that different developmental stages can express different lectins and that even closely related species show differences in lectin expression.

## 3. Some Methodological Considerations to Include in the Study of Lectin Interaction with Pathogens

The classical method to study lectin–pathogen interaction is by isolating them from tissues, blood, or secretes. This often requires a large amount of starting material, and therefore, several studies have used recombinant protein instead of or in addition to isolated protein in functional studies. Making recombinant proteins, if it is possible for the protein of interest, gives the opportunity to obtain relatively large amount of lectins. However, if the lectin under study is toxic to typical genetically modified cells such as bacteria, yeast, or other single cell organisms, finding a working expression system could be time consuming. In addition, an often-neglected point is the fact that many proteins are post-translationally modified and if one uses recombinant proteins there is a need to verify that the recombinant protein is the same as the one produced by the original host. Especially in cases where lectins are undergoing co-translational transport into the rough endoplasmatic recticulum for later secretion or transport to the endosomal/lysosomal system, it is crucial to determine the nature of the N-linked and O-linked glycosylation present. It is important to use a eukaryotic expression system and to verify that the glycosylation pattern is identical to or as similar as possible to the expected one. The use of proteins has huge benefits in that it is possible to genetically change parts of the protein to be able to study activities such as binding specificities. However, the verification that the recombinant protein has the same modifications as those of the in situ protein are often not done in in vitro experiments. When producing biopharmaceuticals, mammalian systems are often used, and measures are implemented to secure correct post-translational modifications [22], such measures also need to be included more often in basic research.

To study the interaction of lectins with pathogens in situ*,* antibodies are useful tools for histological and cell morphological observations. Antibodies made specifically against the lectin from the species studied should ideally be produced. Alternatively, antibodies produced against a lectin from another species could be used. In both cases it is crucial to check the specificity as well as sensitivity of the antibody. This could be done by using western blotting and checking the identity of labelled bands with mass spectrometry [23,24].

## 4. The Role of Mucosal Lectins in Agglutination and Hemagglutination, Binding to Viruses, Bacteria, and Parasites

Agglutination of cells and viruses is a process by which they adhere or clump together. The molecules that induce the adherence are called agglutinins i.e., proteins that lead to agglutination. Antibodies are classical agglutinins, important as part of the adaptive immune system and as natural antibodies in the innate immune system [25]. In the innate part of the immune system, lectins are one of the important agglutinins. Many mucosal lectins agglutinate bacteria and non-self erythrocytes; agglutination of the latter is called hemagglutination. When non-self (viruses, bacteria, yeast, and parasites) bind to lectins it can lead to agglutination, targeting for destruction (through activation of complement or by endocytic/phagocytic uptake), or direct destruction mediated by the lectins. In mucosal surfaces, mechanisms such as agglutination are important in preventing pathogen uptake. In the skin and gill mucus layers, agglutination can let the pathogen fall off during swimming, and in the gastrointestinal system the pathogens can be expelled with the faeces. Mucus contains many immune relevant molecules besides lectins [5,26] and can agglutinate bacteria and non-self erythrocytes. In the mucus, several mucosal proteins could work together in the agglutination process, but in the case of isolated or recombinantly produced lectins, agglutination is observed without adding other proteins, showing that lectins alone can perform agglutination and hemagglutination. Protein conformation is influenced by the ions present, temperature, and pH that specifically influences the agglutination capacity of the proteins. Therefore, heating can inhibit agglutination [27] and agglutination can be Ca^++^ dependent [27,28]. Lectins that only have one carbohydrate recognising domain (CRD) must form dimers or higher-order structures to be able to agglutinate.

Agglutination by mucosal lectins was among others shown for galectin-1 from Atlantic cod [17], galectin AJL-1 from the Japanese eel, [20], galectins congerins from the conger eel (*Conger myriaster*) [29], pufflectin from pufferfish (*Takifugu rubripes*) [11], rOnMBL from Nile tilapia (*Oreochromis niloticus*) [27], SsLTL, lily-type lectin from black rockfish (*Sebastes schlegelii*) [28], CsLTL-1, lily-type lectin from striped murrel, (*Channa striatus*) [30], a C-type lectin from black rockfish (*Sebastes schlegelii*) [31], and C-type lectin AJL-2 from the Japanese eel [19]. Congerin from the skin mucus of the conger eel induce agglutination of *Vibrio anguillarum*, but not growth inhibition or bacterial killing [29]. This shows that binding to a pathogen and the inhibition of growth or killing are not necessarily linked.

To understand lectin–pathogen interaction, the non-self molecule(s) that lectins bind to should be identified. A rhamnose-binding lectin, first isolated from the eggs of the ayu (*Plecoglossus altivelis*), also expressed in mucosal tissues as well as in the gills and intestine, could agglutinate a microsporidian (*Glugea plecoglossi*). To identify the biding partners, rhamnose-binding lectin was screened for binding against a hundred oligosaccarides and found to bind to globotriaosylceramide (Gb3) [32]. However, Gb3 was not found in the microsporidian, and the binding molecule was thus not identified.

Agglutination of yeast can also be induced by lectins; Dln1, a natterin lectin from zebrafish, agglutinates yeast [33].

Pufflectin from pufferfish binds to trematode, *Heterobothrium okamotoi* [11]. This lectin is novel in that it does not share homology with other animal lectins but with mannose-binding lectins from plants. It has a mucosal expression in the gills, oral cavity wall, oesophagus, skin, and one isoform exclusively expressed in the intestine. The mucosal expression suggests a role in upholding the barrier function of these tissues. It could be mainly involved in parasitic defence as it bound the *Heterobothrium okamotoi* trematode, but none of the other several types of bacteria tested [11].

Hemagglutination is also common and were among others shown for mannose-binding SsLTL lily-type lectin from black rockfish (*Sebastes schlegelii*) [28], for mannose-binding natterin-like protein from Atlantic cod (*Gadus morhua*) in an calcium dependent manner [13], and a mannose-binding lectin from flathead (*Platycephalus indicus*) with homology to kallikrein [34].

## 5. The Role of Mucosal Lectins in Growth Inhibition and Killing of Pathogens and Changes in Lectin Expression during Infection

Antibiotic resistance is a serious problem and new cytotoxic molecules are sought after. Several lectins show cytotoxic activity against microorganisms and parasites [35]. Even if lectins have been shown to bind directly and alone to cells to agglutinate, it is also possible, and has been demonstrated in mammalian lectins, that they can act as part of complexes when they serve their biological function.

The fact that lectins can bind to pathogens opens the possibility that they can inhibit growth and/or directly kill them. Unfortunately, assays where one cannot distinguish between growth inhibition and killing are often used to study lectins and the effect is in general referred to as ‘antibacterial’. Growth inhibition is also important; however further studies characterising lectins should aim to establish if killing of pathogens is taking place. This could, for example, be done using fluorescent stains, such as propidium iodide, that only stain cells with holes in the cell membrane.

A spectrophotometer is often used to study antibacterial activity. *E. coli* incubated with C-type lectin AJL-2 from the Japanese eel shows lower absorbance at 600 nm than control samples, indicating growth inhibition or killing of the bacteria [19]. The spectrometric assay can be performed at several wavelengths, but 600 nm is the most common one as interference from common broths is low. The assay measures turbidity, that is the refraction of light brought about by the particles present and it does not separate dead cells from live ones.

Also, clearance zone observation in agar is often used, and a mannose-specific tetrameric lectin from the ovary of the cobia (*Rachycentron canadum*) gives a clearance zone in the agar diffusion test suggesting antibacterial activity or growth inhibition effects against *E. coli* [36].

Natterins are lectins with an N-terminal carbohydrate-binding domain that is jacalin-like that binds to mannose and with a C-terminal aerolysin-like toxin domain. Natterin has been purified from skin mucus of Oriental stinging catfish (*Plotosus lineatus*) [37] and from Atlantic cod [13]. The arolysin-like domain is interesting as its structure suggests it could lyse pathogens. In zebrafish, 16 putative homologs of natterin are found in the genome [38]. Dln1 was studied in detail; it was a dimer in aqueous form and a pore forming octamer when inserted into membranes [33]. Natterin gene (*aep1*) expression increased after the *Staphylococcus aureus* challenge and injection of the protein before the challenge increased the bacterial clearance [39]. *Aep1* was expressed in early developmental stages, before the adaptive immune system is expressed, suggesting it is part of the innate immune system [39].

Lectins also have roles in the protection against viruses; an F-type lectin, RbFTL-3, from rock bream (*Oplegnathus fasciatus*), mainly expressed in the intestine in the animal, could protect fathead minnow cells from hemorrhagic septicemia virus infection in vitro [40]. A proteomics study of RbFTL-3, showed that transfected and hence protected cells, showed interesting changes in proteins related to viral budding (SNF8, CCT5, and TUBB) and in thrombin signaling, compared to control cells [40].

*In vivo* studies of fish challenged with pathogens show changes in gene or protein expression of lectin genes or proteins, suggesting roles in immune defences. The mannose-binding lectin OnMBL’s gene and protein (from Nile tilapia) [27] were up-regulated after challenge with a Gram-positive bacterial pathogen (*Streptococcus agalactiae*) or a Gram-negative bacterial pathogen (*Aeromonas hydrophila*) suggesting a role in the defence against bacteria. The mRNA of a mannose-binding, lilly-type lectin, SsLTL gene was up-regulated in the gill after polyinosinic-polycytidylic acid (poly I:C) and *Streptococcus iniae* challenges, suggesting a role in the defence against viruses and bacteria. Up-regulation, however, was not a general trend as a challenge with lipopolysaccharide down-regulated SsLTL expression, but the down-regulation was minimal compared with the several fold up-regulations observed with poly I:C and *S. iniae* [28]. Fish lily type lectin-1, CsLTL-1 from striped murrel (*Channa striatus*) was up-regulated in the gills after infection with fungus (*Aphanomyces invadans*) and bacteria (*Aeromonas hydrophila*) [30]. Together this shows a diversity of mannose-binding lectins in fish, which can change gene and/or protein expression upon challenge by virus, bacteria, and fungi. This indicates that they have a role in the protection of the host. However, as virus, bacteria, and other pathogens could highjack host machinery to get access to host cells, further experiments are needed to show that an increased amount of lectin is protective for the host and not promoted by the pathogens to increase infection.

Channel catfish (*Ictalurus punctatus*) have twelve galectin genes and most of these are highly expressed in mucosal tissues. Interestingly, the expression profile of galectins showed tissue-type and pathogen-type-specific changes upon challenge with two Gram-negative bacterial pathogens [41].

The galectins congerin I and II from the Japanese conger eel, can bind to *Cucullanus* nematodes that parasitise the abdominal cavity [42]. The encapsulated nematodes were often found to be dead or dying, but some could have also escaped through an unknown mechanism [42]. Galectin-1 from Atlantic cod were also found in granuloma in *Francisella noatunensis* infected fish, where it seemed to be extracellular suggesting a role in opsonisation and/or direct killing of the pathogen [16].

## 6. The Role of Lectins in Chemotaxis

Fish mucus can induce chemotaxis in bacteria. The chemotaxis stimulation by different fish individuals vary and the skin, gill and intestine mucus have different effects. In channel catfish, mucus from the skin and gills were more stimulatory than mucus from the intestine, and skin mucus from 60% of the fish stimulated chemotaxis [43]. In a study of *V. anguillarum*, different serotypes of the bacterium were chemotactic to skin mucus from rainbow trout, Atlantic cod, common bream, and flounder [44]. In a study of gilt-head sea bream (*Sparus aurata* L.), several *Vibrio* strains showed chemotactic activity towards mucus that could be inhibited by high or low salinities and/or temperatures [45]. It has been suggested that bacteria use chemotaxis to find their host, the tissue to infect, and that chemotactic bacteria have higher growth rates in their host than non-chemotactic ones [46].

In a study of *Flavobacterium columnare* it was found that the chemotactic activity of *F. columnare* towards mucus from channel catfish (*Ictalurus punctatus*) was inhibited by some sugars whilst others were not effective in inhibiting [47]. The ability to inhibit the chemotactic activity by sugars strongly suggests that this activity is dependent on lectin(s).

## 7. The Role of Mucosal Lectins in Endocytosis and Phagocytosis

Uptake of pathogens serves two functions in immunoprotection, the first is the destruction of the pathogen, usually in the lysosome, and the second is the sampling of the pathogen so that peptides from the pathogen are presented to the immune system. Lectins are traditionally thought of as a part of the immune system with a role in protecting against diseases; interestingly, lectins can also play a crucial role in promoting infections, and hence help pathogens. It is the role of lectins as opsonines, promoting the phagocytic and endocytic uptake of pathogens, that can be exploited by the pathogens to get access to cells.

An interesting case is that mRNA of rhamnose-binding lectin was found to be upregulated in the gills of catfish (*Ictalurus* spp.) early after *Flavobacterium columnare* infection [48]. *F. columnare* causes columnaris disease and the role of the rhamnose-binding lectin is in promoting infection rather than inhibiting it [49]. Catfish not susceptible to infection by *F. columnare* do not upregulate rhamnose-binding lectin mRNA when exposed to the pathogen, whilst susceptible fish do. Furthermore, in susceptible fish, infection is inhibited by l-rhamnose and d-galactose, putative rhamnose-binding lectin ligands [49]. Rhamnose-binding lectins from chum salmon (*Oncorhynchus keta*) bind to globotriaosylceramide (Gb3) and promote phagocytosis [50]. Gb3 is well known as the receptor for the B subunit of the very potent shiga toxin from *Shigella dysenteriae* 1 and some serogroups of *Escherichia coli*, and it stimulates clathrin dependent endocytic uptake of the toxin [51].

Uptake of viruses and bacteria by endocytosis and phagocytosis can be used for the destruction of the pathogen, and the goal for the host is delivery to the lysosome where the pathogens will be degraded. However, in the tug-of-war between invaders and the immune system during evolution, pathogens have developed means to escape. Some escape from the early endosome/phagosome just after uptake, others escape later, whilst some block fusion with the lysosome or have developed methods to survive in the lysosome [52].

In studies of teleost lectins there has been a focus on the uptake of pathogens. Promotion of uptake has been found for many lectins. The F-type lectin from sea bass (*Dicentrarchus labrax*) expressed and also localised in the liver and intestine, for example, increased fucose-inhibitable and phagocytic uptake of *E. coli* by peritoneal macrophages compared to the control [53]. In head kidney leukocytes from Atlantic cod, galectin-1 seemed to be secreted from cells that had phagocytosed latex beads [16], suggesting that phagocytosis can stimulate the release of lectin to further stimulate phagocytosis during infections.

## 8. Inhibiting Uptake of Pathogens, Inhibiting Colonisation of Surfaces and Inhibiting Biofilm Formation

The interaction of host surface receptors with pathogen ligands can give pathogens entry into cells. Blocking this interaction serves as a defence mechanisms against pathogen invasion and surface colonisation.

Two galectins, Drgal1-L2 and Drgal3-L1 from zebrafish, were effective in binding to infectious hematopoietic necrosis virus (IHNV) glycoprotein. The virus enters the host at the base of the fins. In vitro the galectins prevented the virus from adhering to fish epithelial cells [54] where it usually binds to a fibronectin at the cells surface [55]. Virus uptake can also be stimulated by lectins, for example SsLec1, a C-type lectin from black rockfish (*Sebastes schlegelii*) increases infectious spleen and kidney necrosis virus (ISKNV) copy numbers in black rockfish [31]. Bacteria on the other hand were efficiently phagocytosed and killed by head kidney macrophages in the presence of SsLec1 [31], and expression of SsLec1 gene increased when fish were infected with bacteria [31]. This is a clear example of the dual role of lectins where a virus is using a pathway developed for pathogen uptake and clearance to get access to cells and infect the host.

Interestingly, in mammals, a secreted antibacterial lectin RegIIIγ is essential to separate the microbiota in the small intestine from the intestinal epithelial surface, resulting in a ~50 μm bacteria free zone [56]. Work on the microbiota of mucosal surfaces of fish has increased in recent years, the information obtained has increased the knowledge of fish mucosal surfaces and established teleosts as a model species for increasing the knowledge regarding host–microbe interactions [57].

Isolated galectin AJL-1 from the Japanese eel inhibits biofilm formation by *A. actinomycetemcomitans* strains Y4, ATCC 29523, and ATCC 29524, a bacterium that causes periodontal disease in humans [58]. AJL-1 also inhibits inflammatory cytokines in human endothelial cells. This suggests that the eel galectin can replace or complement antibiotic treatment of periodontal disease in humans [58].

## 9. The Possible Use of Fish Lectins in Human and Veterinary Medicine

It is in principle possible to use lectins to promote animal and human health. Lectins that bind to viruses are potential antiviral drugs. Dln1 a natterin-like protein from zebrafish has a high affinity to gp120 of HIV (human immunodeficiency virus) and it was suggested to be a possible antiviral agent [33]. Lectins that prevent the binding of viruses to cell surfaces inhibit further proliferation of viruses, but care must be taken to ensure that the lectin used do not cause side effects. Dln1 has a pore-forming, aerolysin-like part in addition to the mannan-binding lectin part and this could be problematic if it were to lyse human cells [33]. The ability to lyse cells could on the other hand be beneficial in the treatment of cancer or targeted treatment towards pathogens or parasites. DLn1 [33] and other fish natterins [12,13,59] could, with targeted modification of the lectin part of the molecule, show specificity for pathogen/parasites or cancer cells and open up for potential therapeutic uses.

A proof of concept that teleost lectins can kill human cancer cells comes from a study on European bass (*Dicentrarchus labrax*) fucose-binding lectin (DlFBL). This lectin, when delivered by an adenovirus vector, induces apoptosis in human cells, including hepatocellular carcinoma cell lines Hep3B and BEL-7404, lung cancer cell line A549, and colorectal carcinoma cell line SW480 [60]. Also, Japanese eel lectin 1 induces apoptosis in human cancer cells when delivered through an adenovirus vector [61]. The apoptosis stimulation by lectins in both studies is through the PRMT5-E2F-1 pathway [60,61]. Skin mucus from the Japanese eel can induce mitochondria-mediated apoptosis in human K562 cells in a lactose inhabitable way, indicating that direct administration of lectins can kill cancer cells [62].

The biofilm inhibition potential of teleost lectins was shown for galectin AJL-1 from the Japanese eel [58] and a use in periodontitis treatment was suggested. New molecules with a potential to interact with bacteria, yeast, and parasites to inhibit growth or induce death are needed as resistance towards established drugs is increasing. Fish is by far the largest group of vertebrates with more than 30,000 known species, making fish an attractive target for bioprospecting to find bioactive products. Antibacterial activity against veterinary and/or human pathogens is suggested for some lectins; however, this is an area where additional research is needed.

## 10. Further Perspectives

Most of the current knowledge on fish mucosal lectins interaction with non-self is from studies of lectins isolated from mucus or tissues or recombinantly produced and then incubated in vitro with viruses or non-self cells. There is a clear need for more experiments on whole animals and cell culture experiments to observe interactions with host cells. There is also little information on the function of mucosal lectins in different locations. Because of the teleost whole genome duplication event 250 million years ago [63,64,65] there is a high diversity in the lectins present in teleosts, especially in salmonids where an additional genome duplication occurred [66]. Several questions need answers: Which functions do extracellular lectins have compared to intracellular lectins? Intracellular lectins are found in different compartments; however, their functional roles in these compartments need further clarification. Is it possible to use sugars to inhibit diseases by treating mucosal surfaces with saccharides through bathing (for the skin, gills, and mouth) and through feed (for the gastrointestinal system) in the cases where host lectins or pathogen lectins are important for infective pathogen uptake? In farmed animals such as fish where the feed is formulated and fed in a controlled manner, manipulation of the microbiota [67] and mucosa [68] through the feed are possible. Also, the possible involvement of lectins in regulating the microbiota on mucosal surfaces is an interesting path to explore as it has been done in mammals [56]. Lastly, could the inhibition of biofilms by fish lectins prevent biofilm formation when this is beneficial in human medicine and odontology or in industry facilities? Could fish lectins provide a novel killing mechanism for pathogens or human cancer cells? Through molecular studies of lectins and their interaction with host and non-self molecules, coupled with cell experiments and animal studies these questions shall be answered.
